# Evaluation of a Point-of-Care Multiplex PCR Analyser for Detection of *Babesia gibsoni* in the Canine Population of Hong Kong

**DOI:** 10.3390/vetsci13060548

**Published:** 2026-06-03

**Authors:** See Mun Tsang, Virginia Merino Gutierrez, Jane Yu, Fraser Hill, Ibrahim Elsohaby, Angel Almendros

**Affiliations:** 1 Department of Veterinary Clinical Sciences, Jockey Club College of Veterinary Medicine and Life Sciences, City University of Hong Kong, Hong Kong SAR, China; 2 Veterinary Medical Centre, City University of Hong Kong, Hong Kong SAR, China; 3 Veterinary Diagnostic Laboratory, City University of Hong Kong, Hong Kong SAR, China; 4 Department of Infectious Diseases and Public Health, Jockey Club College of Veterinary Medicine and Life Sciences, City University of Hong Kong, Hong Kong SAR, China; ielsohab@cityu.edu.hk

**Keywords:** *Babesia gibsoni*, canine babesiosis, Hong Kong, point-of-care PCR, validation

## Abstract

Canine babesiosis caused by *Babesia gibsoni* is prevalent in Hong Kong. While molecular diagnosis via PCR is highly sensitive, it typically requires reference laboratory access, delaying clinical decisions. This study evaluated the diagnostic accuracy of a point-of-care multiplex PCR compared to a reference laboratory PCR for detection of *B. gibsoni* in dogs. A retrospective cross-sectional study was conducted on 47 canine EDTA blood samples collected between May 2024 and June 2025. Samples were tested for *B. gibsoni* using a point-of-care multiplex PCR analyser (POCMA) and a reference-standard real-time PCR. Clinicopathological data were evaluated when available. Diagnostic sensitivity, specificity, predictive values, and agreement between tests were calculated. The reference-standard assay detected 23 positives (49%) and 24 negatives (51%) whereas the POCMA detected 20 positives (43%) and 27 negatives (57%). The point-of-care assay demonstrated a sensitivity of 82.6% (95% CI: 62.9–93.0%) and specificity of 95.8% (95% CI: 79.8–99.3%) for *B. gibsoni* detection. All samples were negative for *B. canis* by the POCMA. The point-of-care multiplex PCR analyser (AIMDX1800) offers rapid, specific detection of *B. gibsoni* with excellent specificity but moderate sensitivity. Positive results in clinically compatible cases can guide prompt treatment, while negative results in symptomatic dogs in endemic areas warrant confirmation by reference laboratory PCR. The absence of *B. canis* detection by the POCMA is consistent with previous literature supporting its rarity in Hong Kong.

## Simple Summary

*Babesia gibsoni* is a common blood parasite in Hong Kong dogs that causes fever, anaemia, and thrombocytopenia. While laboratory tests are accurate for diagnosis, they are often slow and expensive, delaying critical treatment. This study tested a new portable, rapid test (AIMDX1800) to assess whether it could provide reliable results in a clinic setting. We examined blood samples from 47 local dogs and found that the test was highly accurate in identifying uninfected dogs (high specificity) but showed also good correlation in detecting infected dogs, although it occasionally failed to detect infections when only low levels of parasite might have been present. In this cohort, mixed-breed dogs were over-represented among the positive cases, though larger studies are needed to confirm breed-related risk patterns. This rapid test supports prompt diagnosis, allowing veterinarians to begin potentially life-saving treatment without delay, although negative results in symptomatic dogs should still be double-checked with a standard laboratory test to ensure accurate diagnosis.

## 1. Introduction

Canine babesiosis is a tick-borne disease of global clinical significance caused by haematoprotozoan parasites of the genus *Babesia.* In Hong Kong, *Babesia* infections are frequently detected in dogs, with prevalence rates ranging from 3.7% to 33% among owned dogs [1,2,3]. Of these cases, *B. gibsoni* accounts for up to 93% of infections [2,3]. While primarily transmitted indirectly via tick vectors such as *Haemaphysalis longicornis* and *Rhipicephalus sanguineus* [4,5], direct transmission can occur through fighting, blood transfusions, or vertical (transplacental and perinatal) routes, making infection possible in regions lacking tick vectors [6,7].

*Babesia gibsoni* infections manifest as either acute or chronic/subclinical disease [7,8]. Acute babesiosis is associated with severe clinical signs including anorexia, lethargy, pale mucous membranes, pigmenturia and fever, alongside frequent haematological abnormalities such as moderate to severe thrombocytopenia and severe anaemia [8]. In contrast, chronic or subclinical infections present with intermittent and more subtle signs [7,8].

Several methods are available for diagnosis of canine babesiosis including light microscopy examination of peripheral blood smears to identify intraerythrocytic piroplasms. Although rapid and specific, this method lacks sensitivity, particularly in chronic cases where parasitaemia is low and organisms may be confused with staining artefacts or other intraerythrocytic inclusions [9,10]. Serological assays, such as immunofluorescent antibody test (IFAT) or enzyme-linked immunosorbent assay (ELISA) are limited by their inability to distinguish between active and past infections; furthermore, they may yield false negatives during acute infection due to the lag-time required for seroconversion [11,12]. Consequently, molecular diagnosis by polymerase chain reaction (PCR) is considered the most reliable method, offering high specificity and sensitivity, even in subclinical or chronic cases [13]. Additionally, PCR enables species identification which has important implications for prognosis and treatment [10]. Despite these advantages, PCR often requires access to reference laboratories, leading to increased costs and logistical delays, potentially hindering timely clinical intervention.

Multiplex PCR point-of-care assays (POCMA) have emerged as a cost-effective method for the detection of multiple pathogens in disease complexes with diverse viral or bacterial aetiologies [14,15]. These assays are particularly valuable when co-infections present with overlapping clinical signs, which is a common challenge in diagnosis of arthropod-borne disease [13,16,17,18]. Given the limitations of current diagnostic tests, a rapid and effective POCMA could significantly enhance the clinical diagnosis of babesiosis. In this study, we assessed the accuracy of an in-house multiplex PCR for the rapid diagnosis of *B. gibsoni* infection in dogs against the validated, gold standard singular PCR at the City University of Hong Kong Veterinary Diagnostic Laboratory (CityU VDL).

## 2. Materials and Methods

### 2.1. Study Design and Sample Selection

A cross-sectional study was conducted using canine EDTA residual blood samples collected from the City University of Hong Kong Veterinary Medical Centre (CityU VMC) and CityU VDL between May 2024 and June 2025. The sample size was determined by the availability of residual specimens (*n* = 47) present during the defined study period. Based on standard sample size calculations for diagnostic accuracy [19], this cohort size is sufficient to evaluate diagnostic performance. Assuming a 1.0 ratio of positive to negative samples, a hypothesised sensitivity and specificity of 95% for the point-of-care PCR assay, and a desired absolute precision (marginal error) of ±15% at a 95% confidence level, a minimum of 17 positive and 17 negative samples was required. Thus, our final cohort (*n* = 23 positives, *n* = 24 negatives) exceeded these minimum thresholds, yielding over 80% statistical power.

The study included 26 residual samples obtained from CityU VMC from client-owned dogs that presented with clinical signs consistent with babesiosis (*n =* 8), asymptomatic dogs undergoing routine screening for haemoparasites (*n* = 2), and dogs presenting for unrelated reasons where tick-borne infection was considered unlikely (*n* = 16). An additional 21 residual samples were obtained from CityU VDL; these had previously tested positive for *B. gibsoni* via a validated simplex PCR assay. These samples originated from cases with a high clinical suspicion of babesiosis submitted by local veterinarians. All samples were anonymized and processed in accordance with ethical guidelines for the use of residual diagnostic specimens, approved by the animal ethics committee of City University of Hong Kong (AN-STA-00000555).

### 2.2. Data Collection

A total of 47 dogs were enrolled in the study. The cohort comprised a range of ages and breeds, representative of the general hospital and referral populations in Hong Kong. Clinical data including age, breed, sex, and presenting history were recorded. For the subset of samples sourced from the CityU VMC (*n* = 26), additional information regarding clinical signs, reasons for the visit, and available haematological and biochemical data were extracted from medical records at the time of the visit when blood was collected for PCR analysis. In contrast, clinical and laboratory data for the 21 samples obtained from VDL were incomplete. Clinicopathological parameters for the CityU VMC subset included haematocrit (HCT), platelet count (PLT), reticulocyte count (RETIC), total protein (TP), and globulin (GLOB) concentration.

### 2.3. Polymerase Chain Reaction (PCR)

A validated, singular PCR assay for *B. gibsoni* performed at CityU VDL was used as the gold standard reference. In comparison, a multiplex point-of-care PCR (POCMA) was used for the simultaneous detection of *B. canis* and *B. gibsoni*.

#### 2.3.1. Reference-Standard Method (CityU VDL)

Approximately 1.3 mL of whole blood samples were collected into EDTA-coated tubes and refrigerated pending subsequent analysis. Detection of tick-borne pathogens was conducted using real-time polymerase chain reaction (PCR), with primer sequences and target genes for *B. gibsoni* (Table 1) [20]. Notably, *B. canis* was not tested at CityU VDL due to its historically low prevalence in Asia.

Genomic DNA was extracted from 200 μL aliquots of each blood sample using an automated platform (EZ1 Advanced XL, Qiagen, Hilden, Germany) according to the manufacturer’s protocol. For *Babesia* detection, DNA extraction was performed using commercial kits (DSP Virus Extraction Kit, Qiagen, Hilden, Germany), which included positive controls. To validate successful DNA extraction, the endogenous housekeeping gene (β-actin) was amplified using specific primers and probes [21]. All *Babesia*-specific primers and the endogenous control primers were synthesised de novo (Techdragon Limited, Hong Kong, China).

PCR amplification was carried out in a final reaction volume containing 10 μL of SYBR Green Supermix (Bio-Rad, Hong Kong, China). The thermal cycling protocol consisted of an initial enzyme activation step at 95 °C for 3 min, followed by 44 cycles of denaturation (95 °C, 15 s), annealing (60 °C, 15 s), and extension (72 °C, 15 s). Fluorescence signals were recorded after each cycle, and samples with a cycle threshold (Ct) value below 40 were classified as *Babesia*-positive. The Ct cut-off values were established during the initial protocol optimisation using the positive control templates. Nuclease-free water was used as a negative control in each run.

#### 2.3.2. Point-of-Care Multiplex PCR Analyser (AIMDX1800)

The AIMDX1800 nucleic acid amplification analyser (Aimbio Hangzhou Zhunxin Biotechnology Co., Ltd., Hangzhou, China) was used with the *B. canis* and *B. gibsoni* nucleic acid detection kit. This assay detects *B. canis* by targeting the heat shock protein 70 (HSP70, 124 bp) and the small subunit ribosomal RNA (SSU rRNA, 162 bp), while *B. gibsoni* is detected via targeting the heat shock protein 70 (HSP70, 92 bp). For internal control, the β-actin (101 bp) protein of *Canis lupus familiaris* is used to ensure assay accuracy and sample integrity.

The system integrates fully automated magnetic bead-based nucleic acid extraction and multiplex real-time PCR into a single, closed-system cartridge (AimCartridge™, Aimbio Hangzhou Zhunxin Biotechnology Co., Ltd., Hangzhou, China). Samples were prepared by adding 200 µL of EDTA whole blood that was loaded into the cartridge after suspending the magnetic beads by inversion. The AIMDX1800 then performs automated lysis, nucleic acid purification, and amplification. Results were displayed and interpreted automatically: positive results were defined as Ct ≤ 36, weak positives as 36 < Ct < 38, and negatives as Ct ≥ 38 or no Ct. The absence of any measurable Ct indicates invalid results due to a lack of internal control amplification, necessitating a retest. For the primary diagnostic accuracy analysis, weak positive results (36 < Ct < 38) were categorised as positive. No invalid results due to internal control amplification failure were encountered in this cohort; therefore, no retesting was required. Had an invalid result occurred, the sample would have been retested once according to the manufacturer’s instructions, and the repeat result used for final analysis.

### 2.4. Statistical Analysis

Statistical analyses were performed using Stata (version 19.5, StataCorp, 2026). Statistical significance was set at *p*-value < 0.05. The diagnostic performance of the POCMA test for detection of *B. gibsoni* DNA in dogs was evaluated using 2 × 2 contingency tables, implemented via the diagt command in Stata. Sensitivity (Se) was defined as the proportion of dogs with *B. gibsoni* infection (as determined by PCR) that tested positive using the POCMA test. Specificity (Sp) was defined as the proportion of dogs without *B. gibsoni* infection that tested negative using the POCMA test. Accuracy was calculated as the proportion of all dogs correctly classified by the POCMA test.

Predictive values depend on test sensitivity, specificity, and disease prevalence in the target population [22]. To evaluate the performance of the POCMA test across populations with varying prevalence of *B. gibsoni* infection, the positive predictive value (PPV) and negative predictive value (NPV) were estimated for hypothetical prevalence values ranging from 1% to 100%. The PPV was defined as the proportion of POCMA-positive dogs that were truly infected, while the NPV was defined as the proportion of POCMA-negative dogs that were truly uninfected.

Agreement between PCR and POCMA results was assessed using McNemar’s test to detect systematic differences between paired results, followed by calculation of Cohen’s kappa statistic to quantify the level of agreement beyond chance [23]. Agreement between the two tests using Cohen’s kappa coefficient, was interpreted as: ≤0.20 = poor, 0.21–0.40 = fair, 0.41–0.60 = moderate, 0.61–0.80 = good, and 0.81–1.00 = very good agreement [24].

For clinicopathological parameters, descriptive statistics were calculated. Continuous variables were reported as median and range due to non-normal distribution. Categorical variables were presented as frequencies and percentages.

## 3. Results

### 3.1. Study Population

A total of 47 dogs were included in the study. The median age was 9 years (range: <1 to 17), with a sex distribution of 57% (27/47) males and 43% (20/47) females. The majority of the dogs in the cohort were neutered (38/47, 81%). Fifteen different breeds were represented, with mixed-breed dogs (18/47, 38.3%) and poodles (9/47, 19.1%) being most common. Followed by Beagle, Bichon Frise, French Bulldog and Miniature Schnauzer (2/47, 4.2% each). The remaining breeds, including Chihuahua, King Charles Spaniel, Labrador, Welsh Corgi, West Highland Terrier, English Springer Spaniel, Shiba Inu, Schnauzer, Pomeranian, Border Collie, Maltese and German Shepherd, each accounted for 2.1% (1/47) of the population. Among the 23 dogs confirmed positive for *B. gibsoni* via the reference-standard PCR, 14 (61%) were of mixed breed, two were Miniature Schnauzers, and the remaining seven represented seven distinct purebreds.

### 3.2. Detection of Babesia Species by PCR

No amplification for *B. canis* was detected across any of the 47 samples by the POCMA. Regarding *B. gibsoni*, the reference-standard PCR detected 23 positive samples (49%), while the POCMA identified 20 positive samples (43%) (Table 2). Concordant results were observed in 42 of 47 samples (89.4%), including 19 true positives (both methods positive) and 23 true negatives (both methods negative). Discordant results were observed in five samples (10.6%) including four false negatives (POCMA negative, CityU VDL positive) and one false positive (POCMA positive, CityU VDL negative) The false positive had a POCMA Ct of 31.81 for *B. gibsoni* but was not detected by the reference assay. Among the four false negative samples, reference PCR Ct values were 39.9, 29.7, 35.4, and 38.1. Two of these four false negatives were follow-up samples from dogs previously treated for babesiosis. All 27 POCMA-negative samples showed Ct ≥ 38 or no Ct with a valid internal control signal. Among the 20 POCMA-positive samples, none fell into the weak positive category (all had Ct ≤ 36). Table 2 presents the 2 × 2 contingency table. In summary, there were 19 true positives, one false positive, four false negatives, and 23 true negatives.

### 3.3. Diagnostic Accuracy of Point-of-Care Multiplex PCR Analyser

**Figure 1:**
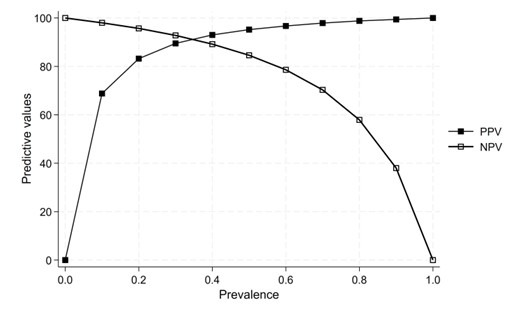
Positive predictive value (PPV) and negative predictive value (NPV) of the POCMA across a hypothetical prevalence (range: 1–100%) of *B. gibsoni*.

Using the reference-standard PCR assay, 23 out of 47 dogs tested positive, representing a reference-PCR-positive proportion of 49.0% within this selected cohort. Compared to the reference-standard PCR, the POCMA demonstrated a Se of 82.6% (19/23; 95% CI: 62.9–93.0%), and a Sp of 95.8% (23/24; 95% CI: 79.8–99.3%). The PPV was 95.0% (19/20; 95% CI: 76.4–99.1%), the NPV was 85.2% (23/27; 95% CI: 67.5–94.1%), and overall diagnostic accuracy was 89.4% (42/47; 95% CI: 77.4–95.4%). At the observed prevalence of 49.0%, the PPV exceeded the NPV, though both remained relatively high (Figure 1).

The overall agreement between the reference PCR and POCMA results for the detection of *B. gibsoni* infection was 89.4%, with a corresponding Cohen’s kappa coefficient of 0.79 (95% CI: 0.610–0.964), indicating substantial agreement. McNemar’s test showed no statistically significant difference between the paired proportions of positive and negative results (*p* = 0.32 and *p* = 0.06, respectively), suggesting no evidence of systematic bias between the two methods.

### 3.4. Clinicopathological Findings

Clinicopathological data were available for 27 of the 47 dogs (57.4%). This subset included all three dogs confirmed positive for *B. gibsoni* by the reference PCR among the CityU VMC-sourced samples, and 24 confirmed negatives. Haematocrit values were recorded for 26 of these 27 dogs, and platelet counts for 26 of 27. The remaining dog lacked both measurements and was excluded from those specific analyses.

Among the 26 dogs with recorded haematocrit, eight (30.8%) were anaemic [HCT < 37.3%, RR 37.3–61.7] [25]. Among the 26 dogs with recorded platelet counts, four (15.4%) were thrombocytopenic [PLT < 148 k/µL, RR 148–484] [26]. Both anaemia and thrombocytopenia were present in two out of the three dogs confirmed positive via the reference-standard PCR. The single false positive case had normal haematocrit (40.7%) and platelet count (260 k/μL).

Among the four false negatives, two were follow-up samples from dogs previously treated for babesiosis, and one had severe anaemia (HCT 13.1%) and thrombocytopenia (PLT 84 k/μL) supporting the true positive result. Two of 24 negative-confirmed dogs (8.3%) had thrombocytopenia (both 0 k/μL), with one having concurrent anaemia suspected to be immune-mediated thrombocytopenia (IMTP). Anaemia was present in four of 24 negative-confirmed dogs (16.7%), which was attributable to onion toxicity (*n* = 1), IMTP with haemorrhage (*n* = 1), chronic non-regenerative anaemia (*n* = 1), and an incidental finding during a neutering presurgical screening in a healthy 8-month poodle (*n* = 1). In all cases where thrombocytopaenia was identified, a blood smear was performed to further assess and support this finding.

## 4. Discussion

This study included samples from 47 dogs of diverse breeds, sizes, sex and neuter statuses. We compared the performance of a POCMA (AIMDX1800) against the reference-standard PCR, using a cohort of 24 negative samples and 23 positive samples. The POCMA demonstrated a Se of 82.6% and a Sp of 95.8% for *B. gibsoni* detection. While the observed Se was lower than the 100% reported in some multiplex PCR validations [17], it remains comparable to, or higher than, other field evaluations where multiplex assays showed reduced Se (40.5–66.7%) compared to singular PCR [16]. The four false negatives (8.5%) may reflect the lower analytical Se often inherent of multiplex PCR, as observed in prior studies [16]. Conversely, the single false positive result (2.1%) could stem from cross-reactivity or low-level parasitaemia below the detection threshold of conventional PCR.

*Babesia canis* was not included in reference-standard testing in this study, and assay validation was therefore limited to *B. gibsoni*. This decision reflects the established epidemiological context in Hong Kong, where *B. gibsoni* is consistently reported as the predominant *Babesia* species in Hong Kong, with prevalence rates ranging from 5 to 45% in local dog populations [1,2,3,8]. The lack of positive *B. canis* POCMA results in our study aligns with regional epidemiological data, suggesting an apparent absence of parasitaemia in the region [2,3]. This observation may be further supported by the fact that natural transmission of *B. canis* depends mainly on the tick vector *Dermacentor reticulatus,* which is not regarded as endemic to Hong Kong or much of Asia [27].

Mixed-breed dogs constituted the majority (61%) of the *B. gibsoni* positive cases, while poodles were primarily found in the negative group (33%). Given our limited sample size (*n* = 47), these breed-specific distributions must be interpreted with caution. However, we cautiously hypothesise that lifestyle factors could influence exposure; for instance, mixed-breed dogs may have a higher likelihood of originating from rescue or stray backgrounds in high-tick burden environments. Conversely, small pure-bred dogs such as poodles might experience more restricted outdoor access [2]. Clinicopathological data was only available for samples collected at CityU VMC where some of the authors practice. Most of the samples from CityU VMC were negative. This might be due to a declining prevalence of the disease in Hong Kong associated with better pet care and the demographic profile of the population presenting at a multispecialty referral centre, which included mainly urban pet dogs regularly treated with preventative ectoparasiticides. The majority of positive-confirmed samples were obtained from CityU VDL as known positives. These lacked clinical and clinicopathological data, which precluded further data analysis in this study. The authors acknowledge that this is not a true representation of the demographics of all dogs in Hong Kong. The main objective of this study was, however, to compare the diagnostic accuracy of a molecular point-of-care test in confirmed positive and negative samples.

The POCMA yielded four false-negative results, including one sample with a reference Ct of 29.7, which would correspond to a moderate parasite burden that would be expected to be readily detectable. The reason for this discrepancy is unclear but may relate to sequence variability in the primer-binding region or to sample degradation. Concurrent anaemia and thrombocytopenia provided clinical corroboration for one of the reference-positive cases; however, the remaining three cases (reference-positive Ct values 39.9, 38.1 and 35.4) lacked sufficient laboratory data for definitive correlation. Notably, two of these discrepant cases (CT 39.9 and 38.1) were follow-up assessments after treatment for babesiosis. It is plausible that post-therapeutic parasitaemia remained present but fell below the analytical limit of detection for the POCMA assay, whereas the more sensitive reference assay successfully identified these low-level infections. Conversely, only a single false-positive result was recorded, involving a neutered male mixed-breed dog. The patient presented with a normal HCT and PLT count, laboratory findings that are inconsistent with acute babesiosis and further support the categorization of this result as a false positive. This false-positive result (POCMA Ct 31.81, reference negative) could represent a true low-level infection below the reference assay’s detection threshold, cross-reactivity, or laboratory contamination.

There were 2 (8%) dogs with thrombocytopenia in the negative-confirmed cases. Both had severe thrombocytopenia (0 k/µL), confirmed on blood smear, and were suspected to have IMTP, with concurrent anaemia in one of the dogs and a normal haematocrit in the other. Such severity of low platelet count is most commonly associated with immune-mediated disease rather than babesiosis [28]. Anaemia was identified in only four (16.6%) of the 24 confirmed negative cases in this study, which was attributable to an alternative aetiology including onion toxicity, concurrent immune-mediated thrombocytopenia (IMTP) with haemorrhage, chronic non-regenerative anaemia, and an incidental finding during presurgical spay screening of a healthy dog that had been on heat. This suggests that the presence of anaemia in *Babesia*-negative dogs should prompt immediate investigation into other common or incidental causes rather than being dismissed as a false-negative result. The authors acknowledge that the reduced amount of clinicopathological data was an important limitation. A further limitation of this study is the absence of PCR testing for *B. canis* using the reference PCR assay. Consequently, negative results obtained with the POCMA should be interpreted with caution.

The sampling strategy may introduce selection bias, as the inclusion of pre-selected *B. gibsoni* positive samples from the reference laboratory artificially enriches the cohort for positive cases and may limit the representativeness of the study population relative to the general canine population or to consecutive clinical presentations. In addition, the overall sample size was relatively small (*n* = 47) and relied entirely on residual diagnostic specimens, further constraining the generalisability of the findings. This approach may also introduce spectrum bias, potentially overrepresenting more severe or clinically suspected cases compared to the broader canine population in Hong Kong. Collectively, these factors should be considered when interpreting the demographic and clinicopathological findings.

In endemic areas such as Hong Kong, anaemia and thrombocytopenia frequently serve as primary clinical indicators for babesiosis testing. Given the high prevalence of the disease in these regions, the integration of point-of-care PCR devices into clinical practice might offer a significant diagnostic advancement. By facilitating rapid, on-site pathogen detection, these tools could allow for the immediate initiation of targeted therapy, potentially improving clinical outcomes. Future large-scale, prospective surveillance studies across diverse clinical settings are needed to minimise these biases and validate our observations.

## 5. Conclusions

The findings of this study indicate that the AIMDX1800 analyser offers a valuable, rapid point-of-care tool for *B. gibsoni* detection, which is particularly beneficial in clinical settings lacking immediate access to specialised laboratories. Given the high specificity observed, a positive result in a dog presenting with compatible clinical signs can be acted upon with confidence, allowing for the prompt initiation of targeted treatment. However, due to the assay’s moderate Se, the device should not be used as the sole method for ruling out infection; negative results in highly symptomatic dogs or highly endemic pockets should be confirmed using reference-laboratory molecular methods. Furthermore, while the absence of *B. canis* detection in this cohort aligns with local clinical expectations, this finding represents device output alone and cannot be used to make definitive recommendations regarding regional *Babesia* species prioritisation without independent reference validation. Future prospective studies utilising a larger, broader clinical cohort are necessary to fully validate this point-of-care platform for both *B. gibsoni* and other haemoparasite species.

## Figures and Tables

**Table 1:** Primers and genes used for the study.

Pathogen	Primer	Gene	Primer Sequence (5′-3′)	Reference
*B. gibsoni*	BG-cox1-F	*B. gibsoni* cox1	CTTCAGCCAATAGCTTTCTGTTTG	[20]
	BG-cox1-R		CCTGAGGCAAGTAAACCAAATAT	

**Table 2:** Comparison of point-of-care multiplex PCR and reference-standard PCR (CityU VDL), including diagnostic performance, for detection of *B. gibsoni* in 47 canine blood samples.

	Reference Standard (+)	Reference Standard (−)	Total	Predictive Values
**POCMA PCR (+)**	19	1	20	PPV: 95%
**POCMA PCR (−)**	4	23	27	NPV: 85.2%
**Total**	23	24	47	
**Diagnostic Accuracy**	Sensitivity 82.6%	Specificity 95.8%		

## Data Availability

The data from this study are available upon request from the corresponding author, in accordance with patient confidentiality and hospital policies.
